# Recent Advances in Fabrication of Durable, Transparent, and Superhydrophobic Surfaces

**DOI:** 10.3390/nano13162359

**Published:** 2023-08-18

**Authors:** Wenxin Luo, Mingjie Li

**Affiliations:** School of Integrated Circuits, Guangdong University of Technology, Guangzhou 510006, China

**Keywords:** transparent, superhydrophobic, roughness, surfaces, durability

## Abstract

Transparent superhydrophobic coatings have been extensively investigated due to their ability to provide self-cleaning properties for outdoor applications. However, the widespread implementation of these coatings on a large scale is impeded by the challenges of poor durability and complex fabrication procedures. In this review, the fundamentals and theories governing the mutually exclusive properties of superhydrophobicity, optical transparency, and susceptibility to wear are introduced, followed by a discussion of representative examples of advanced surface design and processing optimizations. Also, robust evaluation protocols for assessing mechanical and chemical stabilities are briefed and potential research directions are presented. This review can offer the research community a better understanding of durable and transparent superhydrophobic surfaces, thereby facilitating their development for real-world applications.

## 1. Introduction

**Figure 1:**
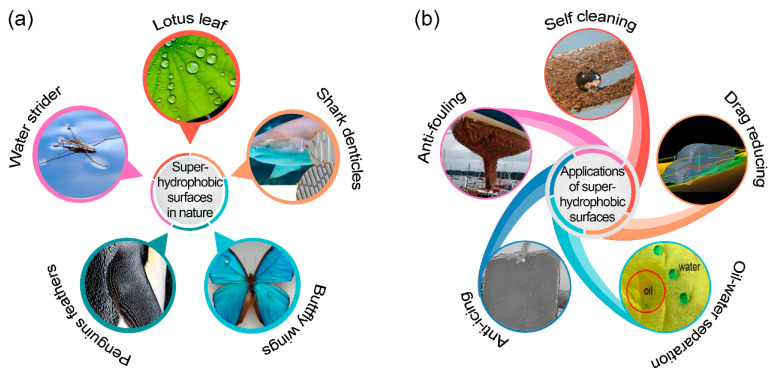
(**a**) Instances of superhydrophobic surfaces found in nature, and (**b**) the corresponding applications.

Inspired by the biological surfaces in nature (Figure 1a), such as the well-known “lotus effect” [1,2,3], superhydrophobic surfaces have attracted considerable research interest across various fields [4,5,6,7,8,9,10,11,12]. These surfaces exhibit exceptional non-wetting capabilities, characterized by a water contact angle of greater than 150° and a water sliding angle (SA) of typically below 10° [13]. The remarkable water-repellent properties of synthetic superhydrophobic surfaces are achieved through a combination of multiscale surface roughness and low surface energy [2,14,15,16]. An increase in surface roughness may lead to a greater contact angle [17,18], while surface energy determines the chemical bonding between liquid and solid atoms. The presence of numerous air pockets trapped between the rough asperities creates a Cassie-Baxter state [19], reducing the solid-liquid contact area and impeding droplet spreading on the solid surface. This unique topography imparts a variety of valuable characteristics, including self-cleaning [9,20], droplet transporting [21], anti-icing [22,23,24,25], anti-fouling [26], drag reduction [27,28], and anti-corrosion [29] (Figure 1b). These advantageous functionalities offer significant potential for engineering applications, such as microfluidic platform, anti-icing airplane surfaces, oil-water separation membrane, etc.

High transparency and superhydrophobicity are often considered mutually exclusive properties for most surfaces. Since the development of superhydrophobic coatings that possess transparency can expand the optical application scope, including self-cleaning windows, electronic screens, windshields, and solar panel covers [12,30], it is crucial to maintain high optical transparency in superhydrophobic surfaces to advance the progress of optical and electronic devices [31,32]. Achieving optical transparency requires the surface material to allow light transmission without significant light scattering. This could be challenging due to the rough surface features, which may lead to light scattering and result in translucency caused by Mie scattering [17,31]. Thus, it is vital to determine the “critical window” where the surface maintains high optical quality in the visible light range while also being water-repellent. Several reviews have extensively discussed transparent and superhydrophobic surfaces, primarily in the context of self-cleaning solar applications [33,34,35]. Yet, despite a few studies that have reported durability enhancement in superhydrophobic surfaces [36,37], there remains a gap in knowledge specifically focusing on the fabrication techniques of durable, transparent, and superhydrophobic coatings. This review aims to bridge this knowledge gap by elucidating the fundamentals and prerequisites governing the conflicting properties of superhydrophobicity, optical transparency, and mechanical durability. Additionally, we present case studies highlighting various durable and transparent superhydrophobic surfaces, which involve top-down, bottom-up, and armor protection techniques. The primary objective of this review is to enhance readers’ understanding of robust self-cleaning and transparent surfaces, thereby stimulating further research in this domain.

## 2. Superhydrophobic Surfaces

### 2.1. Contact Angle on Solid Surfaces

Wettability refers to the capacity of a liquid to retain contact with a solid, resulting from solid-liquid-air interactions. Various parameters are employed to evaluate wettability, such as shedding angle, sliding angle, contact angle, and contact angle hysteresis, whereas contact angle is the most decisive factor. The equilibrium contact angle within a given system of solid, liquid, and vapor is specific to certain temperature and pressure conditions. A large contact angle indicates reduced contact area between the spherical droplet and the solid surface, signifying poor wetting. Conversely, a small contact angle denotes complete wetting, with the liquid evenly distributed over the surface. Based on the contact angle, wetting behavior may be categorized into four distinct regimes, namely superhydrophilic, hydrophilic, hydrophobic, and superhydrophobic (corresponds to unfavorable water wetting behavior). These regimes are characterized by contact angles falling within the ranges of 0°<θ<10°, 10°<θ<90°, 90°<θ<150°, and 150°<θ<180°, respectively. The evaluation of surface wettability using contact angle measurements is a widely utilized method due to its convenience. However, several factors can impact the accuracy of these measurements. These factors include the size and weight of the liquid droplet, the height from which the drop is deposited onto the substrate, and the selection of the baseline at the contact surface [38]. To guarantee unbiased and reliable measurements, it is imperative to employ standardized operating procedures and adhere to universal guidelines.

### 2.2. Classic Wetting Models

**Figure 2:**
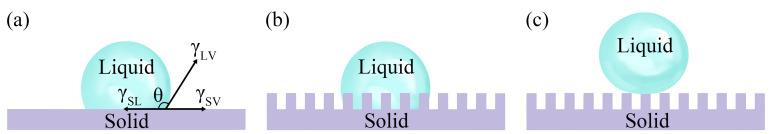
Schematic illustrations of classic wetting models: (**a**) Young’s model, (**b**) Wenzel model, and (**c**) Cassie-Baxter model.

To gain a thorough comprehension of superhydrophobicity, it is necessary to understand the interplay among surface energy, roughness, and wettability. Numerous classic wetting models, including Young’s [39], Wenzel’s [40], and Cassie-Baxter’s model [19], have been proposed to calculate the contact angle on solid surfaces, as depicted in Figure 2.

The wettability of liquid droplets on solid surfaces depends on the equilibrium between gravity and the surface free energy of the interfaces involved, namely solid-vapor (SV), solid-liquid (SL), and liquid-vapor (LV). This thermodynamic equilibrium may be described by Young’s model [39]:

(1) $$\sum F=\gamma_{SV}-\gamma_{SL}-\gamma_{LV}\cos\theta =0$$

where θ is the Young’s contact angle. This model disregards surface roughness when describing the behavior of a liquid droplet on a chemically uniform and ideal smooth solid surface. The theoretical equilibrium relationship between the intrinsic water contact angle and surface energies of each interface in plane can be rearranged as

(2) cos⁡θ=γSV−γSLγLV

In the case where θ=0°, liquid spreading is observed, leading to the formation of a thin film of liquid. If θ<90°, wetting is favorable, while wetting is not favorable when θ>90°, indicating that the liquid tends to exhibit a reduced contact area with the solid surface.

In practice, the majority of surfaces display roughness and irregularities, which significantly influence the wetting properties. Consequently, the applicability of Young’s model for evaluation is compromised. To address this limitation, Wenzel’s model [40] is proposed. This model assumes that a liquid droplet fully contacts the surface and has the ability to completely wet the small furrows of the solid surface. The model also presumes chemical homogeneity throughout the hard surface. The Wenzel contact angle ($\cos\theta_{r}^{w}$) is mathematically expressed as

(3) $$\cos\theta_{r}^{w}=r\cos\theta_{0}$$

where r is the roughness factor defined as the ratio between the actual contact area between a droplet and a surface and the projected area from the top. $\theta_{0}$ represents the contact angle on a perfectly smooth surface with identical surface chemical properties. It is noteworthy that r is usually greater than 1, resulting in an amplification effect on the rough surface. For example, if $\theta_{0}>90{}^{\circ}$, then $\theta_{r}^{w}>\theta_{0}$.

However, the Wenzel model may be inadequate in certain cases due to the inherent inhomogeneity of real surfaces. The Cassie-Baxter model [19] proposes that a liquid drop is supported by a composite surface consisting of two components rather than being completely submerged by surface irregularities. With increasing surface roughness, the sagging of liquid is hindered by air pockets trapped within the surface grooves, leading to limited contact between the liquid droplet and the solid surface at the peaks of the roughness [13]. As a result, the presence of these trapped air pockets causes the surface to become non-wettable. The ideal Cassie state ($\theta_{r}^{c}$) can be defined as follows:

(4) $$\cos\theta_{r}^{c}=f_{1}\cos\theta_{1}+f_{2}\cos\theta_{2}$$

where $f_{i}$, $\theta_{i}$ represent the fraction of the interfacial contact area and contact angle of liquid-solid and liquid-air, respectively. Assuming that air is completely non-wettable and $\theta_{2}=180{}^{\circ}$, the modified Cassie state can be rewritten as

(5) $$\cos\theta_{r}^{c}=f_{1}\left(1+\cos\theta_{1}\right)-1$$

Based on the theoretical Cassie-Baxter model, there is a significant increase in $\theta_{r}^{c}$ as $f_{1}$ decreases. Furthermore, several advanced models have been developed to address specific wetting conditions, aiming to offer more precise predictions and consider the distinctive attributes of wetting scenarios [38].

## 3. Transparency, Durability, and Superhydrophobicity

**Figure 3:**
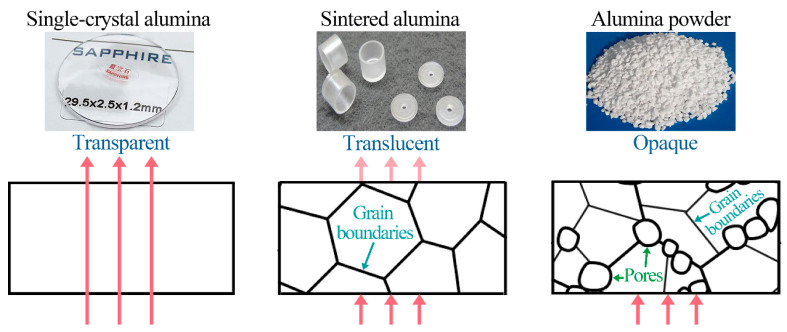
Photographs and schematic illustrations depicting various forms of optical transparency exhibited by alumina with diverse crystal structures.

Transparency enables the passage of light through a material without significant scattering. However, the presence of roughness, pores, and grain boundaries can greatly compromise transparency by causing visible light scattering [31]. As shown in Figure 3, single crystal alumina (e.g., sapphire), which lacks grain boundaries, allows the majority of light to pass through. Thus, it is commonly utilized for camera lenses and wristwatch cases. In contrast, sintered alumina products or alumina powders exhibit grain boundaries and/or pores between the initial powder grains, rendering them translucent or opaque due to pronounced reflections and scattering.

The primary cause of translucency resulting from roughness is the Mie scattering effect [17,41] between surfaces and air. Mie theory assumes that surface roughness can be represented by non-absorbing spherical particles, capable of deflecting incident light and causing a loss in transparency [42,43]. According to the Mie scattering theory, the scattering cross-section, which quantifies light scattering, is proportional to the square of the particle diameter. Surface features such as roughness may induce significant light scattering. Thus, the occurrence of surface roughness, which enhances water repellency, inversely affects optical transparency. Superhydrophobicity and transparency are considered conflicting surface properties. Existing methods for preparing superhydrophobic coatings often lack transparency and/or durability, thus limiting their potential optical applications.

By considering Mie scattering, particle size or surface roughness must be smaller than the wavelength of incident light to achieve high visible light transparency. Previous studies have shown that sub-100 nm roughness is the optimal range for achieving superhydrophobic surfaces with outstanding optical transparency [44,45], extending the boundaries of the transparency-superhydrophobicity trade-off. However, the rough micro/nano-structures required for superhydrophobicity are susceptible to abrasion, posing durability challenges. During abrasion, only a minor portion of rough asperities comes into contact with the abrasive object, generating high local stresses and vulnerability to wear. Consequently, the main obstacles to advancing superhydrophobic surfaces are the inherent trade-off between durability and superhydrophobic properties, as well as the light scattering issues associated with the required rough surface features. While researchers have achieved artificial water-repellent coatings with excellent transparency, mechanical durability, substrate adhesion, and chemical robustness individually, simultaneously demonstrating all these features remains a challenge.

## 4. Fabrication of Transparent Superhydrophobic Surface

### 4.1. Top-Down Method

Effective surface roughness can be achieved by creating micro/nanopatterns on the substrate or surface of interest through both “top-down” and “bottom-up” approaches [38], followed by surface modification to endow low surface energy [16]. Common top-down techniques include femtosecond laser ablation [46,47,48,49], electrodeposition [10], etching [18,38], lithography [50], imprinting [51,52], and the like. In the top-down fabrication process, the initial rough structure becomes an integral component of the underlying substrate, providing it with relatively high mechanical robustness. The physical properties of the substrate or bulk material are crucial in determining the final quality of the superhydrophobic surface.

**Figure 4:**
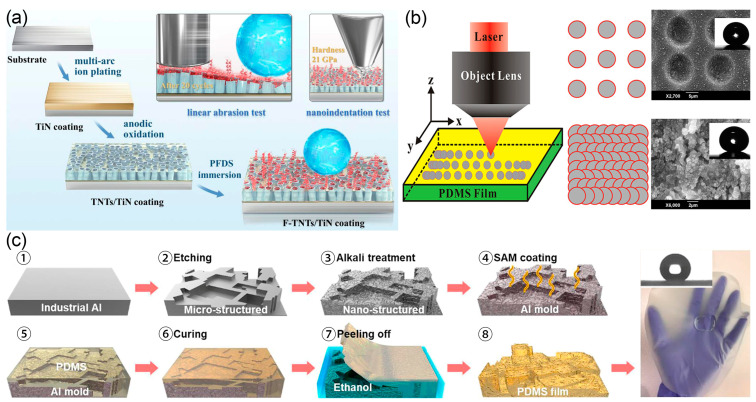
Schematic illustrations of the fabrication process of (**a**) durable transparent superhydrophobic F-TNTs/TiN composite coatings [53], (**b**) PDMS microwell arrays with different period (or overlapping) [54], and (**c**) PDMS film replicated and detached from a two-tier hierarchically structured aluminum mold [55].

Gao et al. [53] reported a robust, transparent, and superhydrophobic fluorinated TiO_2_ nanotube (F-TNTs)/TiN coating, fabricated through surface structure tuning of TiN (Figure 4a). A composite structure was created by depositing an ultra-thin TiN coating using multi-arc ion plating, followed by anodic oxidation to form the embedded TiO_2_ nanotube arrays. Low surface energy was achieved by treating the surface with 1H,1H,2H,2H-perfluorodecyltriethoxysilane (PFDS). The top two-thirds of the layered coating consisted of porous layers with embedded TiO_2_, while the lower layer exhibited a dense TiN structure. The rigid coating yielded moderate optical transparency (visible light transmittance of 79.6%) and a water contact angle of ~154°, demonstrating remarkable self-cleaning capability. Moreover, the layered composite coating displayed a nanoindentation hardness of 21.7 GPa and maintained excellent hydrophobicity after 20 cycles of sandpaper abrasion under a load of 100 g. This design presents an innovative concept for durable nanocomposite coatings. However, such water-repellent surfaces may not meet the requirements for accommodating non-flat objects.

There is a pressing need for the development of a flexible superhydrophobic surface that can easily integrate with complex geometries, while maintaining satisfactory durability to withstand significant deformations. Yong et al. [54] introduced a novel one-step approach to produce superhydrophobic surfaces that offers a high level of control over liquid adhesion. This approach utilized polydimethylsiloxane (PDMS) microwell arrays (Figure 4b). The microwell arrays were swiftly created using a femtosecond laser scanning process. The resulting superhydrophobic surfaces exhibited adjustable water adhesion through modifying the degree of overlap between adjacent microwells. However, the fabrication procedure was associated with low throughput and high cost, rendering it the primary bottleneck that restricts its practical applicability.

In order to reduce manufacturing costs, alternative methods have been reported for the creation of flexible and transparent superhydrophobic surfaces. For instance, transparent and superhydrophobic films were produced by replicating inexpensive PDMS patterns from an aluminum template in a nondestructive and repetitive manner [55] (Figure 4c). The micro/nano structures on the reusable aluminum plate were created through consecutive etching, followed by alkali treatment. The resulting films exhibited superior water repellency without the need for additional surface modifications, because of the hierarchical surface structures and inherent hydrophobicity of PDMS. These superhydrophobic films demonstrated good mechanical flexibility and durability while maintaining their anti-wetting properties even under dynamic deformation/loading conditions, such as mechanical pushing, bending, and stretching. To facilitate the clean release of replicas on a large scale without damaging the expensive master mold, a low-surface-tension liquid infiltration method was employed for film detachment. This approach is scalable, cost-effective, and suitable for a wide range of applications that require multifaceted films with desirable mechanical flexibility and optical properties.

Similarly, Yu et al. [56] successfully developed flexible, transparent, and stretchable PDMS micro-architectures with mushroom-like structures using a series of modified photolithography and thermal treatment techniques. The negative mold with mushroom structures was obtained by employing different baking temperatures in multiple spin-coated photoresist (PR) layers, followed by development. The PDMS mixture was then carefully spin-coated and cured onto the negative PR mold to achieve the PDMS mushroom structures. To enhance the superhydrophobic properties, nanostructures were coated on the PDMS mushroom structures via chemical vapor deposition of nano-SiO_2_, followed by calcination and silane treatment. The resulting hybrid film exhibited excellent stability during different mechanical durability tests. The usefulness of the superhydrophobic film was demonstrated as a protective layer for solar modules and surveillance cameras.

**Figure 5:**
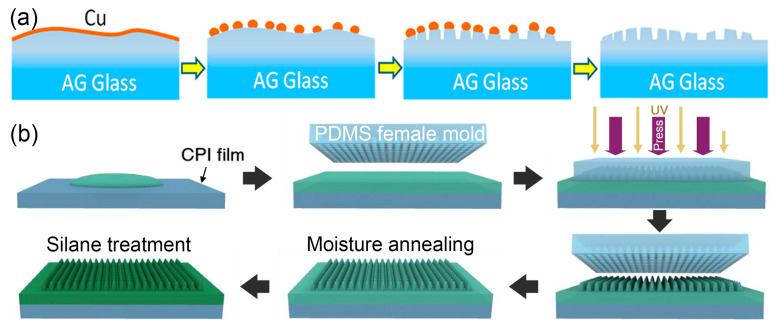
Preparation procedures of (**a**) surface moth-eye nanostructures integrated in the AG glass: Cu thin film and de-wetting are utilized to produce metal nanoparticles hard mask, followed by subsequent dry etching to create the nanopillars on the substrate surface [59]; (**b**) moth-eye structured epoxy–siloxane molecular hybrid (ME-ESMH) on a colorless polyimide (CPI) film through UV-NIL [60]. Orange dots indicate de-wetted Cu thin film.

In recent years, there has been a significant increase in the interest surrounding biomimetic subwavelength structures, particularly due to the remarkable performance exhibited by moth-eye (ME) structures [57,58]. Subwavelength nanopillars exhibit minimal light scattering, thereby enabling directional transmission of beams and ensuring surface clarity. Hierarchical micro-nanostructured glass surfaces possess both superhydrophobic characteristics and desirable optical properties. Tulli et al. [59] utilized reactive ion etching through a thermally de-wetted nano-mask to fabricate monolithic nanostructures (orange dots in Figure 5a). This nano-mask was conformally deposited onto a micro-structured antiglare (AG) glass surface, creating a two-tier hierarchical roughness geometry that exhibits superior water-repellent properties. Additionally, the glass composition was carefully designed to facilitate simultaneous ion exchange of the substrate, micro-structured surface, and nanostructured surface, resulting in enhanced mechanical strength. The resulting glass substrate possessed antireflective and superhydrophobic properties, making it suitable for high-transmission displays with broad-band low reflectivity.

More recently, the utilization of the ME structure has paved the way for research in the development of cost-effective, large-area superhydrophobic coatings through the integration of nanoimprint lithography (NIL). Yun et al. [60] introduced a new class of ME-ESMH on a CPI film. This structure was fabricated utilizing a mass-producible UV-NIL method [61,62], employing a hard PDMS mold replicated from a nickel master template (Figure 5b). The resulting structure exhibited low reflection, superhydrophobic characteristics, excellent inward foldability, as well as remarkable chemical and thermal resistance. Moreover, in situ uniaxial compression tests demonstrated that the surface may be elastically deformed and almost restored to its initial shape even after substantial compression. These findings presented a versatile and practical solution for integrating flexible superhydrophobic coatings onto diverse substrates, particularly for applications in foldable optoelectronics.

### 4.2. Bottom-Up Technique

In contrast to top-down methods, bottom-up techniques involve the coating or hydrothermal growth of nanoscale components with the required roughness, such as nanoparticles, nanowires, nanorods, or nano-clusters, onto the surfaces of interest [38,63,64,65], making them more cost-effective for large-scale production. The externally grown rough micro/nanostructures generated through bottom-up approaches are not integrated with the substrate or bulk material. Consequently, the durability of the resulting coatings becomes a challenge.

SiO_2_ nanoparticles are widely utilized owing to their facile synthesis and the ability to tailor properties like particle size and surface functionality [45,66,67,68]. Commercial SiO_2_ nanoparticles with high visible transmittance are also available at affordable prices [32]. The blending of SiO_2_ nanoparticles with a hydrophobic polymeric precursor offers a viable method for producing superhydrophobic nanocomposite coatings given their simplicity and cost-effectiveness [63,65,68,69,70,71,72]. The mixture can be sprayed [44,69,73,74,75], dip-coated [76,77], spin-coated [68], or deposited via the sonication technique [67] onto various targeted substrates, resulting in a transparent superhydrophobic coating. These coatings can be engineered to exhibit self-similar failure characteristics, where the texture and functionality of the exposed areas after damage closely resemble those of the undamaged layer [78]. Their durability in coatings arises from their ability to maintain superhydrophobic properties through a sequential removal of layers with similar characteristics. As a result, these coatings are resistant to high temperatures, UV light irradiation [73], tape peeling [68], and mechanical bending [44]. In most cases, SiO_2_ particles are incorporated in modified epoxy nanocomposites with a weight percentage ranging between approximately 20 wt.% and 50 wt.% [65,79]. Whereas a higher percentage of silica content can negatively impact film formability [79], optical transparency [38,65], and coating adhesion. Therefore, proper control of SiO_2_ particle content is crucial.

**Figure 6:**
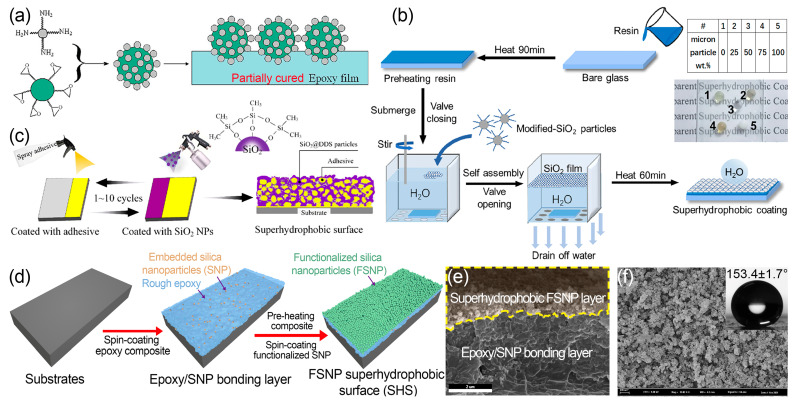
Preparation of superhydrophobic films based on (**a**) SiO_2_ raspberry-like particles [81]; (**b**) self-assembly of SiO_2_ micro/nanoparticles in aqueous medium [82]; and (**c**) adhesive and hydrophobic paint [7]. (**d**) Fabrication of a double-layer designed transparent superhydrophobic nanocomposite coating, and corresponding (**e**) cross-sectional and (**f**) top SEM images [83].

In addition, epoxy resin [2,80,81], cyclic olefin polymer [8], or other commercial adhesives [7] could serve as effective bonding layers between particles and substrates. Ming et al. [81] achieved superhydrophobicity by partially embedding a single layer of two-tier- structured SiO_2_ particles on a partially cured epoxy film (Figure 6a). However, their fabrication procedures involve tedious chemical treatments and could be time-consuming, limiting their applicability to large-scale manufacturing. Similarly, Lyu et al. [82] presented a distinctive aqueous self-assembly method for creating superhydrophobic coatings, utilizing octyltrimethoxysilane-modified SiO_2_ particles and hydrophobic interactions (Figure 6b). These particles were dispersed in water and then self-assembled to form a film. After removing the water, the resulting self-assembled film was deposited on a glass slide coated with semi-cured epoxy resin. This approach produced coatings with transmittance of 94.2% compared to pure glass. The coatings also yielded remarkable resistance against dynamic impact, long-lasting corrosion, and UV illumination, ensuring structural stability. In another work, Chen et al. [7] presented a scalable approach to fabricating environmentally friendly and mechanically durable superhydrophobic surfaces (Figure 6c). They constructed a ferroconcrete-structured superhydrophobic coating by consecutively and repetitively spraying a commercial adhesive from 3 M and an ethanol suspension of modified SiO_2_ nanoparticles. This preparation strategy was applicable for both rigid and flexible substrates. The coated surfaces demonstrated exceptional superhydrophobic behavior with minimal water adhesion (water contact angle of 160°). Moreover, the coated surface retained its superhydrophobicity even after undergoing 325 cycles of linear abrasion using sandpaper under a load of 100 g, demonstrating outstanding mechanical durability.

Inspired by pioneer research, we have recently developed an inexpensive and straightforward method that utilizes spin-coating to fabricate double-layered superhydrophobic surfaces. The upper layer consisted of hydrophobic SiO_2_, which provides the nanoscale roughness, while the lower layer was a rough epoxy/SiO_2_ bonding layer [83] (Figure 6d). The technique involved attaching modified nanoparticles onto a partially cured nanoparticle–epoxy layer (Figure 6e,f). The double-layered coating structure exhibited textural roughness, demonstrating remarkable optical transparency of 97.6% and superhydrophobicity. Additionally, this coating exhibited excellent mechanical durability, chemical robustness, anti-UV function, and thermal stability. The compact surface configuration and flexibility of the bonding layer also contributed to high water impact resistance. By combining these appealing properties, this approach offered a practical and viable solution for transparent applications in outdoor optical devices.

**Figure 7:**
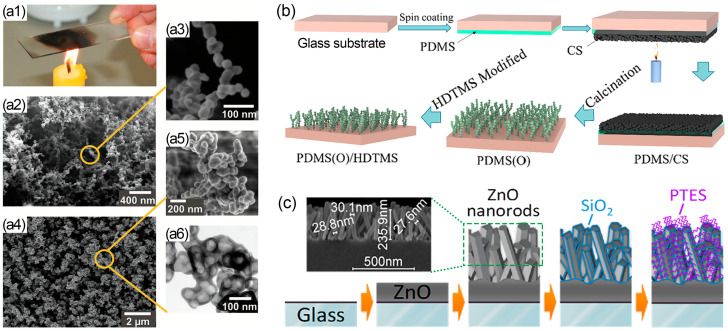
(**a1**) Photograph depicting candle soot preparation. SEM images of the carbon beads deposit network (**a2**,**a3**) before and (**a4**) after being coated with a SiO_2_ shell. (**a5**) SEM and (**a6**) TEM images of a cluster of SiO_2_ shell after carbon soot was removed by calcination [84]. Schematic diagram illustrating (**b**) the fabrication procedure of the oxidized PDMS/HDTMS coating, and (**c**) the formation of SiO_2_-coated ZnO nanorod arrays on glass substrate [86].

Candle soot is a black byproduct that results from incomplete combustion during the burning of paraffin candles. It primarily consists of black carbon particles with an average diameter ranging from 30 to 40 nm, forming a loosely connected, fractal-like network. Due to its easy accessibility, low surface energy, and non-toxicity, candle soot has garnered significant attention for constructing superhydrophobic surfaces. However, candle soot is delicate due to weak particle–particle interactions and is susceptible to damage from water flows or even slight finger touch. To overcome these limitations, researchers have developed methods to reinforce the desirable hierarchical structure of candle soot and achieve a transparent superhydrophobic surface. Deng et al. [84] deposited a 25 nm thick SiO_2_ shell on candle soot using chemical vapor deposition of tetraethoxysilane. They then removed the soot template through calcination to obtain a transparent superhydrophobic and superoleophobic surface (Figure 7a). More recently, Xiao et al. [85] developed a method to create a durable transparent superhydrophobic coating with oxidized PDMS and hexadecyltrimethoxysilane (HDTMS) using morphology of soot particles as a sacrificial template (Figure 7b). Initially, a porous network of candle soot was deposited on a partially cured PDMS coating. After calcination treatment, the candle soot was removed, leaving behind a fully oxidized PDMS layer that inherited the hierarchical structure. The resulting coating exhibited an optical transparency of approximately 88.1%. Further coating with a HDTMS layer produced a superhydrophobic coating with a water contact angle of 163°. These prepared coatings not only delayed the freezing time of water droplets but also facilitated ice to slide away by gravity.

Other metal oxides, such as ZnO and indium tin oxide (ITO) [31,76], have been extensively investigated due to their high hardness, remarkable chemical and thermal stability, high transmittance, and low parasitic reflectance of visible light [87]. Gao et al. [86] reported the development of a superhydrophobic coating based on ZnO nanorod arrays coated with SiO_2_/perfluorodecyltriethoxysilane (Figure 7c). This coating exhibited exceptional transparency (with an average optical transmittance ranging from 93% to 95%) and demonstrated superior UV resistance. The process for preparing this coating involved sequential low-temperature (<150 °C) steps, which were applicable to both glass and flexible polyethylene terephthalate (PET) sheets. Consequently, water contact angles of 157° and 160° were achieved on glass and PET substrates, respectively. The incorporation of SiO_2_ layers on the ZnO nanorod surface effectively provided protection against photo-oxidation reactions induced by UV radiation. Moreover, Ebert and Bhushan [76] utilized a versatile dip coating technique involving SiO_2_, ZnO, and ITO nanoparticles, which were hydrophobized with octadecylphosphonic acid, along with silicone resin, to create transparent, superhydrophobic surfaces on various substrates. These surfaces demonstrated resistance to sliding wear when tested against an atomic force microscopy (AFM) tip owing to the strong bonding between the silicone resin and the nanoparticles, as well as the sufficient hardness of both components.

### 4.3. Robust Armor Strategy

Previous research on top-down and bottom-up approaches has encountered a dilemma: the delicate nanostructure tends to cause weak mechanical durability due to lack of strong protection [22,88,89]. Therefore, it remains a considerable task to simultaneously maintain mechanical robustness, transparency, and hydrophobic properties of the coating through a cost-effective technique.

Wang et al. [90] demonstrated liquid-repellent surfaces composed of micro-structured frames containing hydrophobic materials within the frame cavities. They employed photolithography and wet etching to create inverted pyramidal micro-cavities on silicon, which were then transferred onto hot-pressed glass substrates. Subsequently, these micropatterns were filled with the Ultra-Ever Dry commercial coating through spraying. However, the replication process of the micropatterns via hot pressing is typically time-consuming and may lead to low preparation efficiency. The interconnected surface frame functions as an ‘armor’, shielding the nanostructures from removal by abradants larger than the frame size. The resulting surface exhibited remarkable durability, withstanding over 1000 cycles of sandpaper abrasion (ten times higher than that of traditional surfaces). When applied to a solar cell, the water-repellent armored coating contributed to maintaining high energy conversion efficiency by passively preventing dust accumulation. This outcome could potentially result in significant savings in freshwater consumption, labor, and other costs. The generality and effectiveness of this multiscale armor concept can offer valuable guidance for future research and investigations on superhydrophobic surfaces.

**Figure 8:**
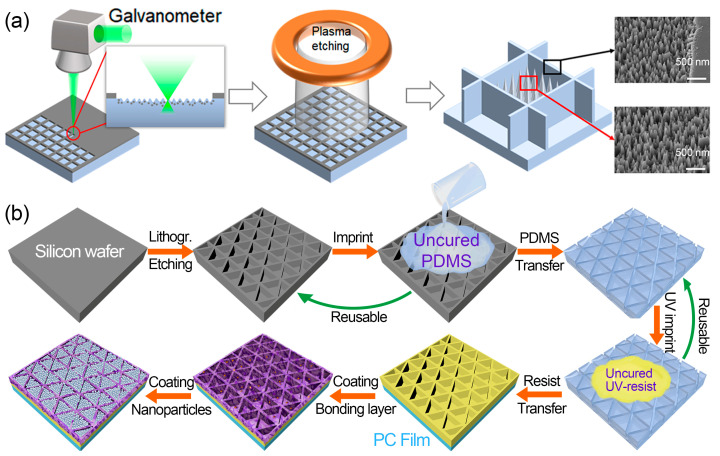
(**a**) Schematics illustrating the fabrication using SDEM and plasma dry etching [91]. (**b**) Preparation of low-cost and scalable transparent superhydrophobic coating [92].

Based on this progress, Xu et al. [91] introduced a novel manufacturing method utilizing the selectively laser-doping-enhanced micromasking (SDEM) effect to prepare a rigid fused silica surface (Figure 8a). This approach involved the creation of robust superhydrophobic structures consisting of locally engraved nanocone arrays with micro-grid walls. The fabrication process utilized an ultrafast laser beam to selectively ablate and induce doping of metals from the deposited hard mask, thereby reinforcing self-assembled nanoparticles that served as additional hard masks during plasma etching. Subsequent etching generated sub-plane engraved subwavelength nanocone arrays in the laser-ablated region. The untreated metal grids acted as hard masks for the protective armors. A chemical vapor deposition process involving fluorosilane was then employed to impart low surface energy and water repellency, providing self-cleaning characteristics. The prepared coatings exhibited remarkable durability with minimal deterioration in superhydrophobicity and transmittance after linear abrasion tests, showing their potential for use on outdoor optical windows of various devices.

Recently, a transparent, durable, and superhydrophobic inorganic coating was successfully produced on a glass substrate using a two-step process involving phase separation and chemical vapor deposition [93]. Initially, the process employed the phase separation approach based on an epoxy resin and acidic silica sol system to create a wear-resistant, porous film on the glass surface. This film featured honeycomb-shaped pores, serving as a micro-scale armor structure. Subsequently, SiO_2_ nanoparticles were applied to the porous cavities using the chemical vapor deposition method, resulting in the formation of a hierarchical structure. Finally, the superhydrophobic film was modified with fluoroalkylsilane to achieve a low surface energy and a water contact angle of 154°. The resulting multifunctional surface exhibited high transparency with excellent visible light transmittance and low haze, demonstrating exceptional self-cleaning performance and resistance to acid corrosion. This new method was not only facile but also suitable for large-scale manufacturing. However, the lack of flexibility in glass or fused silica substrates presented a challenge for integrating these techniques with non-planar objects.

Motivated by the exemplary design concept of previous research, we developed an ingenious method to construct a highly durable and flexible superhydrophobic coating (Figure 8b) [92]. The coatings consisted of a multilayer structure, comprising a top layer of fluorinated SiO_2_ nanoparticles, which enhanced surface roughness and water repellency, and a bottom layer of UV-cured resist frame arranged in a triangular micro-wall pattern. The layers were held together by a rough epoxy-nanoparticle adhesive layer. The proposed coating yielded a water contact angle of 153.6° and low sliding angle of 3.2°. Notably, the micropatterned multilayer surface demonstrated robust resistance to abrasion from sandpaper while maintaining its desirable hydrophobic properties. This outstanding performance can be attributed to the effective protection provided by the rigid architecture and the good adhesion stability of the bonding layer. Moreover, the prepared surface maintained excellent water repellency even when subjected to water jet impact, acid submergence, and mechanical bending, indicating its ability to sustain superhydrophobic properties under adverse conditions and its compatibility with non-flat geometries. Additionally, the superhydrophobic coating exhibited anti-reflective characteristics while retaining optical transparency. These remarkable properties made our superhydrophobic coating highly appealing for scalable manufacturing in various outdoor applications, including self-cleaning covers for solar cells.

## 5. Durability Evaluation Protocols

The preceding sections primarily discuss the preparation of durable and transparent superhydrophobic surfaces. The variation in fabrication strategies also results in differences in the stability of superhydrophobic surfaces. Even for identical superhydrophobic surfaces, their durability may vary when subjected to different external damage stimuli. Researchers often employ diverse evaluation methods, yet with varying setups, to investigate the mechanical and chemical stability of transparent superhydrophobic surfaces, making cross-literature comparison difficult. Despite outstanding research works, it is necessary to establish universal testing standards and protocols for evaluating the durability of superhydrophobic coatings.

**Figure 9:**
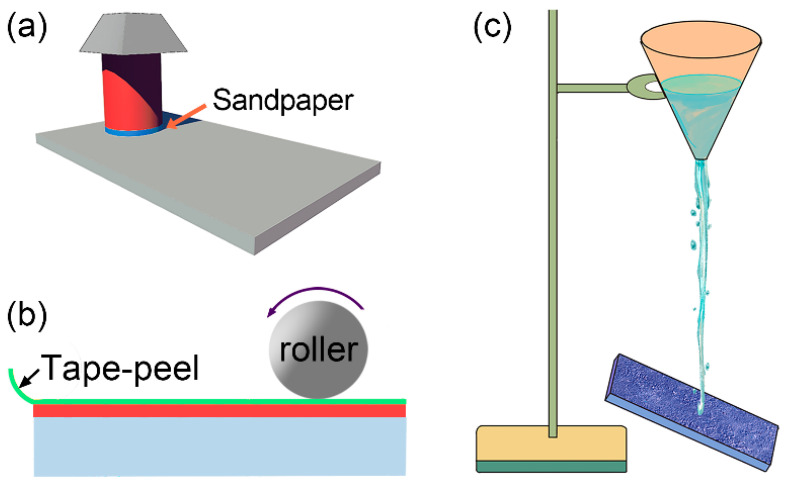
Schematic setup for (**a**) linear abrasion, (**b**) tape peeling, and (**c**) water impact test.

According to Cassie’s model, the prerequisites for achieving superhydrophobicity are a rough hierarchical structure and low surface energy. However, the delicate rough asperities may be compromised by linear and rotary abrasion, tape-peeling, and sand and water jet impact testing (Figure 9). The linear abrasion test involves repetitively moving the superhydrophobic surface along the same contact line against an abradant to simulate possible mechanical damage. Abrasion conditions are typically quantified by adjusting the load, abrasive size, and abrasion distance while simultaneously monitoring the evolution of water repellency. Similarly, the rotating abrasion test utilizes a Taber abrasion machine with two loaded abrasive wheels, where superhydrophobic surfaces are rubbed on a rotary platform. The tape-peeling test measures the adhesion stability of the filling nanomaterials on the substrate. The peeling resistance of the surface is typically inferred from the correlation between tape peeling cycles and the resulting water contact angle. Additionally, external mechanical impacts (e.g., water jet) and elevated hydrostatic pressure can compress the air pockets within the rough structures, leading to a transition from the metastable Cassie state to a Cassie-Wenzel partial wetting state. The impact resistance of the superhydrophobic surface is usually evaluated by monitoring changes in the surface contact angle in relation to the impact height, velocity, total mass of impurities, and number of cycles. Moreover, superhydrophobic surfaces are widely recognized as effective solutions for mitigating icing. The durability of icephobicity on superhydrophobic surfaces may be assessed through cyclic icing and de-icing processes, where ice is either naturally or mechanically removed [94].

The low-surface-energy films may react with corrosive media, which can be assessed by using different pH solutions and immersion times to evaluate surface chemical stability. The selection of entirely organic constituents contributes to enhanced chemical robustness [78]. Natural aging can be tested by subjecting the surfaces to UV irradiation and a temperature-controlled environment to mimic aging conditions. The exceptional chemical inertness and high temperature resistance of superhydrophobic coatings with anti-icing performance are highly desirable. The durability and versatility of transparent water-repellent surfaces, when applied to various substrates, are essential for the long-term success of coating applications. To facilitate a more intuitive comparison of the durability of transparent superhydrophobic surfaces prepared using different fabrication strategies, their stabilities are summarized in Table 1.

## 6. Conclusions

Transparent superhydrophobic surfaces with mechanical robustness and broad suitability play a crucial role in long-lasting coating applications. In this contribution, we first briefly highlight the importance of developing nature-inspired transparent and superhydrophobic surfaces for various potential applications. Subsequently, the fundamentals of superhydrophobicity and classical wetting models are introduced, along with an exploration of the conflicting relationship between water repellency, transparency, and durability. Furthermore, case studies of cutting-edge experimental designs and fabrication strategies for transparent superhydrophobic surfaces, including top-down, bottom-up, and multiscale armor protection techniques, are discussed. The preparation methods that exhibit wide adaptability, as well as eco-friendly and cost-efficient coating capability, are essential for scalable manufacturing. Commonly employed approaches for assessing durability are also summarized. This introductory review aims to provide the audience with a concise understanding of the focused area and help to inspire advancements in robust, transparent, and superhydrophobic coatings for future practical applications.

## Figures and Tables

**Table 1:** Comparison of the mechanical and chemical durability of the literature-reported transparent superhydrophobic surfaces.

Method		Surface	Transp.	Abrasion Resistance	Tape Peel	Water/Sand Impact	Chemical Stability	Flexibility	Ref.
Top-down	Anodic oxidation	F-TNTs/TiN	80%	CA of 144° after 400 cm sandpaper abrasion under 1.7 kPa	CA of 143° after 100 cycles		CA of 145° after immersion of 1 M HCl solution for 4.5 h	Non-flexible	[53]
	Pattern replication	PDMS	76%	CA of 151° after 5 cm sandpaper abrasion under 1 N	CA of 148° after 20 cycles	CA of 157° after 20 g sand impact from 50 cm height		CA of 165° under 30% strain	[56]
	Pattern replication	PDMS	92%			CA of 152° after 20 g sand impact from 20 cm height	CA of 152° after UV irradiation for 7 d	CA of 153° after 10,000 cycles of bending	[55]
Bottom-up	Soot template	PDMS(O), HDTMS	88%	CA of 145° after 1000 cm sandpaper abrasion under 1 N	CA of 150° after 40 cycles	CA of 145° after water impact from 23 cm height for 50,000 drops	CA > 150° after acidic (pH1) immersion for 170 h		[85]
	Spin-coating	SiO_2_, GPDF	93%	CA of 128° after 1000 cm sandpaper abrasion under 0.5 N	CA of 140° after 200 cycles	CA of 135° after water impact from 50 cm height for 6000 drops	CA of 145° after HCl (0.1 M) immersion for 40 min		[63]
	Spin-coating	SiO_2_, oligomer	85%		CA > 150° after 30 cycles	CA > 150° after water jet at 100 kPa for 3 min	CA > 150° after acidic (pH2) immersion for 24 h	CA > 150° after 50 cycles bending	[68]
	Spin-coating	SiO_2_, epoxy	89%		CA of 145° after 50 cycles	CA of 153° after water jet at 1 m/s for 35 min	CA of 150° after 10% HCl immersion for 200 min	CA of 150° after 1000 cycles bending	[83]
	Self-assembly	SiO_2_, epoxy	83%		CA < 150° after 10 cycles	CA < 150° after water jet at 8.6 m/s for 6 min	CA < 150° after H_2_SO_4_ (5 M) immersion for 55 h		[82]
Armor	Hot pressing, spraying	Glass, Ultra-Ever Dry	92%	CA > 150° after 1000 cycles sandpaper abrasion under 3 N	CA > 150° after 100 cycles	CA > 150° after 400 mL water jet at 20.4 m/s	CA < 150° after NaOH (2.5 M) immersion for 4 h	Non-flexible	[90]
	Imprint, spin-coating	SiO_2_, epoxy, resist	92%	CA of 133° after 128 cm sandpaper abrasion under 26 kPa		CA of 150°after water jet at 1 m/s for 35 min	CA of 150° after 10% HCl immersion for 200 min	CA of 150° after 1000 cycles bending	[92]
	Spin-coating, CVD	SiO_2_, epoxy	89%	CA of 131° after 2000 cycles flannel abrasion under 30 kPa	ISO 2409-1992 (0–1 grade)		CA > 150° after H_2_SO_4_ (pH1) and NaOH (pH14) immersion for 48 h		[93]

## Data Availability

No new data were created or analyzed in this study. Data sharing is not applicable to this article.
